# Monitoring brain activity and behaviour in freely moving *Drosophila* larvae using bioluminescence

**DOI:** 10.1038/s41598-018-27043-7

**Published:** 2018-06-18

**Authors:** Manuela Marescotti, Konstantinos Lagogiannis, Barbara Webb, R. Wayne Davies, J. Douglas Armstrong

**Affiliations:** 1 Brainwave-Discovery Ltd., Edinburgh, Scotland UK; 2 The University of Edinburgh, Edinburgh, Scotland UK; 3 Centre Of Developmental Neuroscience, King’s College London, London, UK

## Abstract

We present a bioluminescence method, based on the calcium-reporter Aequorin (AEQ), that exploits targeted transgenic expression patterns to identify activity of specific neural groups in the larval *Drosophila* nervous system. We first refine, for intact but constrained larva, the choice of Aequorin transgene and method of delivery of the co-factor coelenterazine and assay the luminescence signal produced for different neural expression patterns and concentrations of co-factor, using standard photo-counting techniques. We then develop an apparatus that allows simultaneous measurement of this neural signal while video recording the crawling path of an unconstrained animal. The setup also enables delivery and measurement of an olfactory cue (CO_2_) and we demonstrate the ability to record synchronized changes in Kenyon cell activity and crawling speed caused by the stimulus. Our approach is thus shown to be an effective and affordable method for studying the neural basis of behavior in *Drosophila* larvae.

## Introduction

Monitoring neural activity is crucial to understanding the underlying mechanisms of animal behavior. Ideally, the neural signals should be measured while the animal is freely behaving, allowing the relationship to be directly explored. *Drosophila* larvae possess a relatively compact nervous system of the order of just 10,000 neurons^1^ and a behavioural repertoire that responds adaptively to a wide range of sensory stimuli^2^. Moreover, transgenic manipulation of the organism is highly advanced with a wide range of targeting systems available^3^. Combined, these features make *Drosophila* larvae an attractive research tool for dissecting the fundamental principles of structure/function relationships in the brain.

Calcium-imaging approaches have been employed to visualize neural activity with increasing spatial and temporal resolution^4,5^. However, such solutions rely on custom arrangements of costly confocal microscopes^6,7^, and/or are limited to either immobilized preparations or partially dissected ones^5,8^. Currently, the preparation associated with confocal imaging requires restraining the larva. This can be a delicate and time-consuming operation that may impact observations, as there is evidence to suggest that neural activity is highly dependent on the behavioral state. For example, spike recordings of head-restrained rats, where whisking was inhibited, were found to be more similar to those seen in anaesthetized rats, rather than recordings from awake animals^9,10^. More generally, constraining the animal limits the possibilities of directly monitoring the correlation of neural activity and behaviour, so the connection between the neural states and the actions of the animal can only be indirectly inferred. We therefore argue that new insights could be drawn from experimental paradigms if these were supplemented with the ability to monitor neural activity and behaviour simultaneously, while the animal is behaving as naturally as possible^11^.

Bioluminescence-based approaches have been used to measure calcium activity in a wide range of *Drosophila* tissues including adult brains^12,13^, and more recently in other species such as zebrafish larvae^11^, where it was used as a surrogate measure of neural activity. In comparison to brain-imaging methods, bioluminescence, measured using photon-counting techniques, is not significantly sensitive to the animal’s movements^11^, and, since it does not suffer from photobleaching^7,11^, it allows for longer term recordings. On the other hand, photon-counting does not easily permit spatial discrimination of the source of the emitted signal (i.e. identity of the active neurons), a problem that will be compounded if the animal is freely moving. Determining identity is therefore usually achieved through genetic targeting of the expression of bioluminescence in specific neurons.

The bioluminescence method used here involves the combined delivery of the apo-aequorin (AEQ) protein (transgenic) and its chemical (pharmacological) co-factor coelenterazine (CTZ)^11^. The Aequorin-based emitted bioluminescence is related to the amount of Ca^++^ bound to AEQ^14^ since, upon binding of oxygen and calcium ions, the reconstituted Aequorin fusion protein releases its cofactor and emits photons in the blue range^15^. Specifically, we utilized a GFP variant of AEQ, and targeted the expression of the GFP-apo-Aequorin encoding construct to neurons of interest. We then measured the emitted photons using standard photon counting techniques, by aiming a photo-multiplier tube (PMT) at the whole specimen^11^.

In developing this method, we took an incremental approach. We began with recording bioluminescence from neural populations in unstimulated and restricted animals, before we progressed to recording from freely crawling larvae under the influence of a controlled stimulus. For this later part, we designed and built a complete apparatus for monitoring both neural activity and behavior, utilizing widely available 3D printing technology and standard components. The system we present is thus a highly affordable setup that can be customized to experiments with different requirements on sensors and stimulants.

## Results

### Spontaneous activity of Kenyon cells can be recorded from intact *Drosophila* larvae

Our initial efforts focused on detecting neural activity from intact but partially constrained larvae. Prior studies employing transgenic AEQ in *Drosophila* adult neural tissues focused on the Kenyon cells (KCs) in the mushroom bodies (MB) of the adult brain^16^. There, a strong spontaneous oscillatory activity^16^ was reported from an adult brain *ex-vivo* preparation. Given that this is a relatively large neural population and that its dense neuropil is implicated in a range of learning-related processes^17,18^, we thought it made for a good starting point and so we set out to replicate these findings using the apparatus shown in Supplementary Fig. 1. We obtained a range of *Aequorin* transgenes from community stock centres and tested them under a variety of conditions and with several CTZ variants and delivery methods. Subsequently, we tested the signal to noise ratio. The highest signal to noise ratio was obtained using a combination of a transgene encoding an Aequorin fusion protein^14^ and an aqueous form of CTZ amenable to delivery in the larval food^18,19^. A range of CTZ concentrations was tested in food, where 12.5 μM appeared to provide enough CTZ to KCs for this type of experiment. Details of the Method and supporting information are provided in the supplementary material.

**Figure 1:**
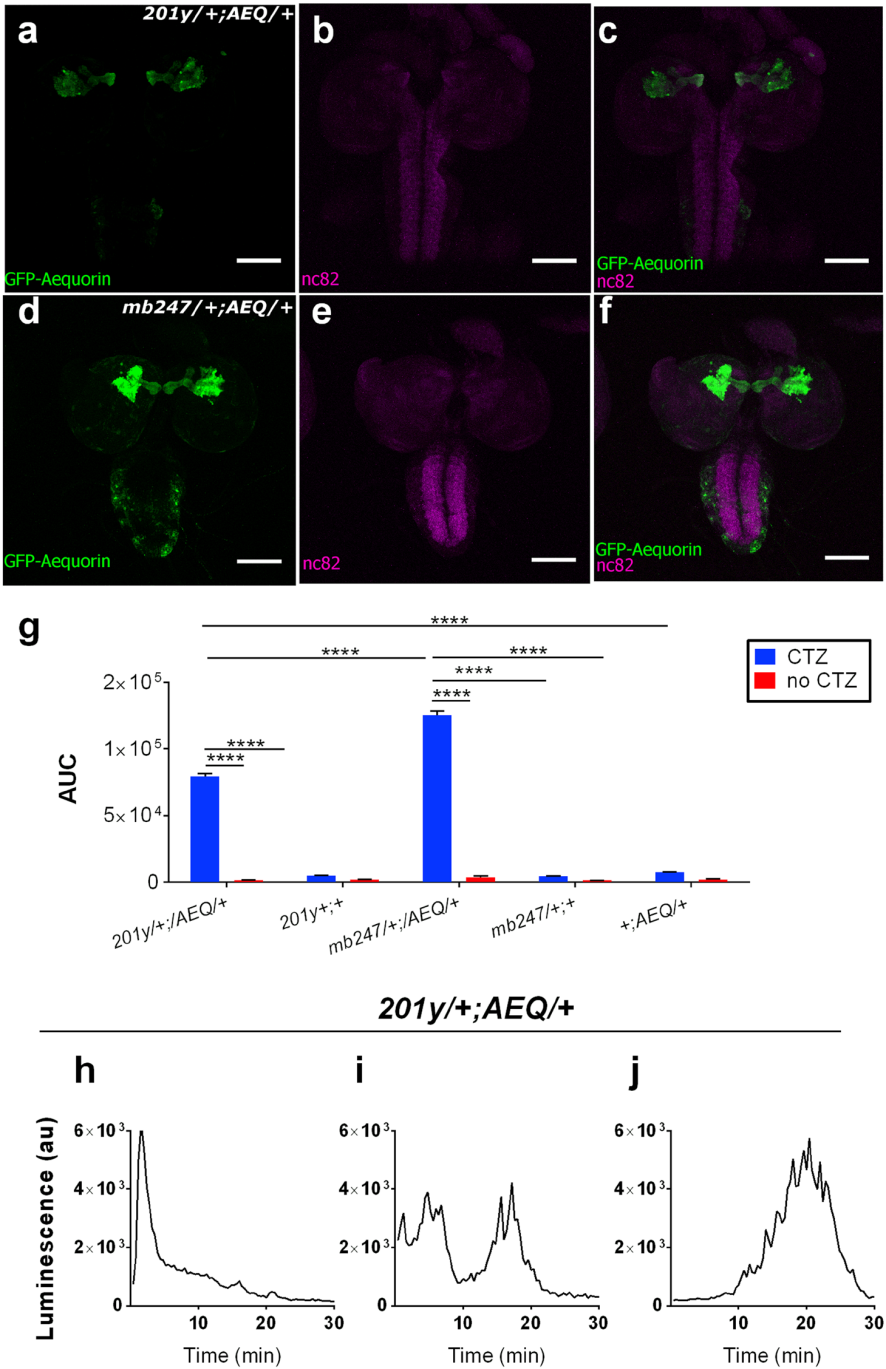
Expression patterns of two Kenyon cell-Gal4 drivers and spontaneous neural activity from larval KCs. **(a**–**c)** 201y and **(d**–**f)** mb247 were crossed, alternatively, with UAS-*GFP-aequorin* and double stained by anti-GFP (green) and nc82 (neuropile marker; magenta). Scale bar represents 100 μm. The bioluminescence-based analysis of these 201y- and mb247- targeted neurons shows a specific range of basal activity that is distinguishable from the control-treatment groups. **(g**,**h)** 30 minutes of bioluminescence signal recorded from *201y/*+; *AEQ/*+ (n = 28, μ = 1,065.989 arbitrary units (au) ±57.085 au), *mb247/*+; *AEQ/*+ (n = 18, μ = 1,630.574 au ±189.542), *201y* */*+; + (n = 18, μ = 68.327 au ±1.821 au), *mb247/*+; + (n = 18, μ = 58 au, ±2 au), +; *AEQ/*+ (n = 20, μ = 48.32 au, ±1.092 au) treatment groups fed with CTZ. In addition, the bioluminescence signal for 30 minutes from all the above named strains in the absence of CTZ is reported where the bioluminescence does not exceed a μ = 22.14 au ±0.456 au (*201y* */*+; *AEQ/*+, n = 21). Sidak’s multiple comparison test was performed between the *201y* */*+; *AEQ/*+ and its genetic controls, and between *mb247/*+; *AEQ/*+ and its genetic controls for both CTZ and no-CTZ conditions. Only significant differences have been shown. Representative traces from *201y/*+; *AEQ/*+ larvae showing variable activity (**i**–**k**). Bioluminescence frame length = 2,000 ms. ****p < *0.0001*.

We crossed AEQ with two larval MB drivers^20^ and showed that we can get a specific signal when expressing in KCs (100 s of neurons) (Fig. 1a–h). In all recordings, we observe transient peaks of activity lasting a few minutes (Fig. 1i–k and Supplementary Fig. 2b), but beside this, the patterns over time are rather idiosyncratic to individuals and we do not see oscillations routinely as previously reported for adult brains^15^. However, differences in MB patterns of activity are to be expected given that the study in question was on an *ex-vivo* preparation and at a different stage of development.

A range of negative controls are presented in Fig. 1g,h and Supplementary Fig. 2a,c, all of which demonstrate that the signal we see is specific and allows the sensitivity to be estimated. The control-experiments consist of: bioluminescence-recordings from fly strains having either the driver (*201y/*+;+ and *mb247/*+;+) or the transgene (+; *AEQ/*+) in the genome; analysis of the same signal from larvae of these genotypes not fed with CTZ; larvae that specifically were not expressing AEQ in MBs (*mb247-gal4-UAS-Tub-gal80ts/*+; *AEQ/*+).

### Bioluminescence depends on the number of neurons expressing Aequorin and the amount of Coelenterazine

Having achieved recordings of bioluminescence signals from a large population of neurons, the larval KCs, we proceeded to test the method on smaller neural groups. First we targeted a reduced KC “model” using the  *201y/*+; *AEQ/*+ strain, which targets fewer neurons and is more specific to the MB than *mb247*. We performed an additional round of optimization via a systematic exploration of parameters (copy number of the driver, copy number of the transgene, incubation temperature of the GAL4/UAS system, CTZ dosage) that could potentially impact the emitted bioluminescence. Our results reveal that the amount of emitted bioluminescence is related to the CTZ dose (Supplementary Fig. 3).

**Figure 2:**
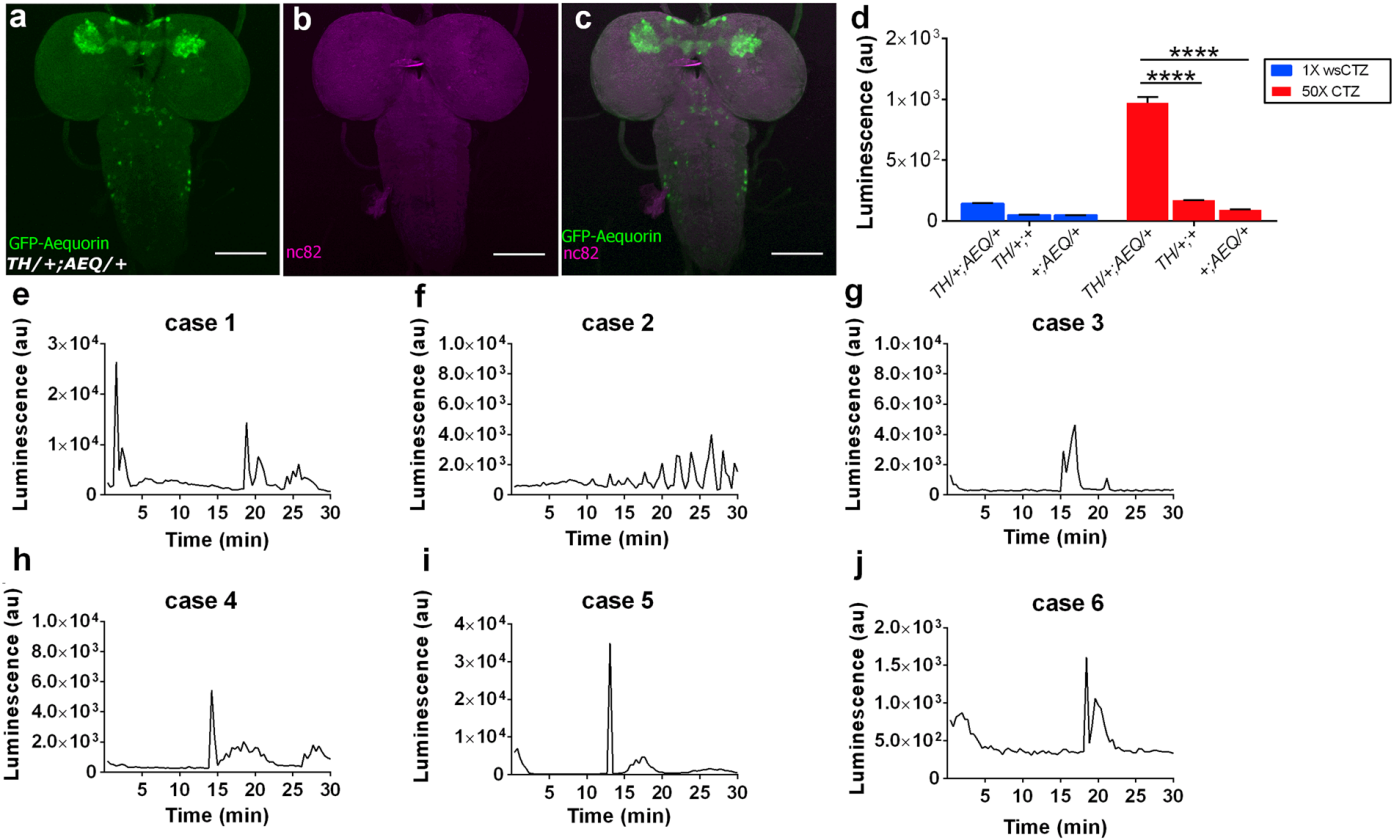
Expression patterns of TH-targeted Dopaminergic neurons and bioluminescence signal amplitude depends on the CTZ concentration. **(a**–**c)** TH crossed with UAS-*GFP-aequorin* and double stained by anti-GFP (green) and nc82 (neuropile marker; magenta). Scale bar represents 100 μm. **(d)** Mean signal recorded over 30 minutes from *TH/*+; *AEQ/*+ (n = 34, μ = 143.71 au ±2.043 au), *TH/*+; + (n = 6, μ = 49.94 au ±1.55 au), and +; *AEQ/*+ (n = 20, μ = 48.31) larvae using 12.5 μM. The same data and analysis are reported for *TH/*+; *AEQ/*+ (n = 13, μ = 969.231 au ±49.538 au), *TH/*+; + (n = 6, μ = 170.911 au ±1.477 au), and +;*AEQ/*+ (n = 5, μ = au ± 1.418 au) larvae incubated in 625 μM CTZ food. Sidak’s multiple comparison test was performed between the *TH/*+; *AEQ/*+ and the two genetic controls for both 1×CTZ and 50×CTZ group of samples. **(e–j)** Representative traces showing the luminescence emitted from *TH/*+; *AEQ/*+ larvae fed with 625 μM CTZ. (****P < 0.0001). Bioluminescence frame length = 2,000 ms.

We further reduced the number of neurons, first by attempting to detect spontaneous activity from the dopaminergic (DA) neurons of the larval brain (90 neurons)^21^, using the Tyrosine-Hydroxylase (TH) driver (Fig. 2a–c), then tested a yet smaller group of DA neurons targeted by the *R58E02* driver (6 neurons in total)^22^ (Supplementary Fig. 4a–e). We managed to obtain a signal from both *TH/*+; *AEQ/*+ and the *R58E02/*+; *AEQ/*+ drivers by increasing the CTZ dose to 625 μM, which is increased  by ×50 compared to the one used in our KC-targeting protocol (Fig. 2d–j and Supplementary Fig. 4d,e).

We performed 30 min. continuous recording for each sample and obtained signals that display sharp peaks of activity, usually between long quiescent intervals. We suspect the differences in the timing of peak onset among samples reflects spontaneous activity from different individual animals (Fig. 2d–j).

### Bioluminescence reveals the neural activity in freely-behaving *Drosophila* larvae

**Figure 3:**
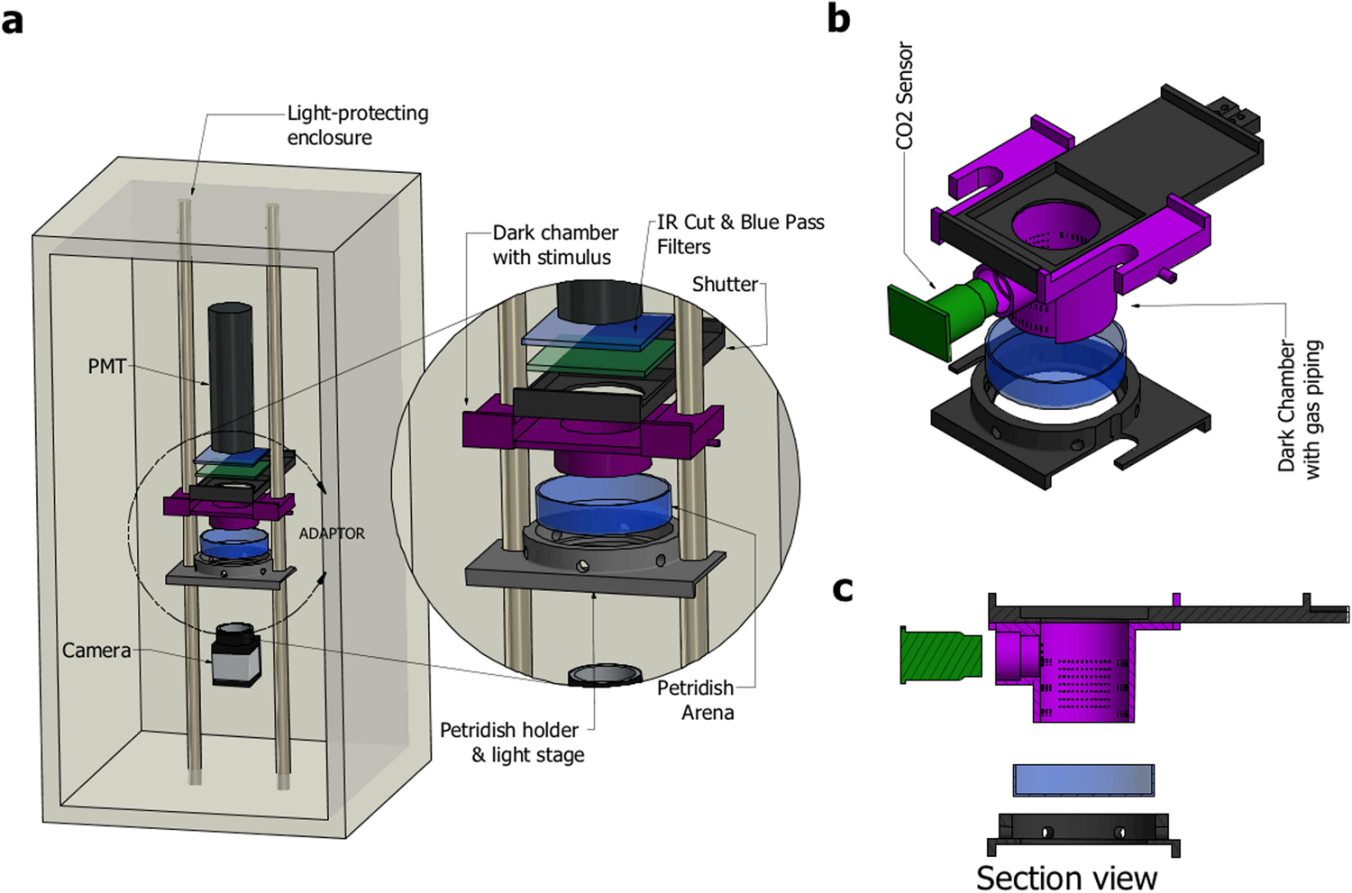
Overview of the designed apparatus for simultaneous bioluminescence and behavioural recordings. (**a**) A light-proof enclosure contains the main components of the apparatus: a PMT, a custom-made adaptor, a petri dish arena with an illumination stage, and a camera. The adaptor sits in front of the PMT, and acts as an optical shutter and a dark chamber. The chamber confines the larva to a 30 mm-diameter arena and allows stimulus delivery. A larva is placed on a 60-mm-dish, which is set on an IR illumination-stage and pushed against the chamber. IR LEDs illuminate the petridish from the side. (**b**,**c)** Adaptor design specific to CO_2_ stimulation. It has a mount point for a CO_2_ sensor and a two-channel gas piping system allowing diffusive air flow.

**Figure 4:**
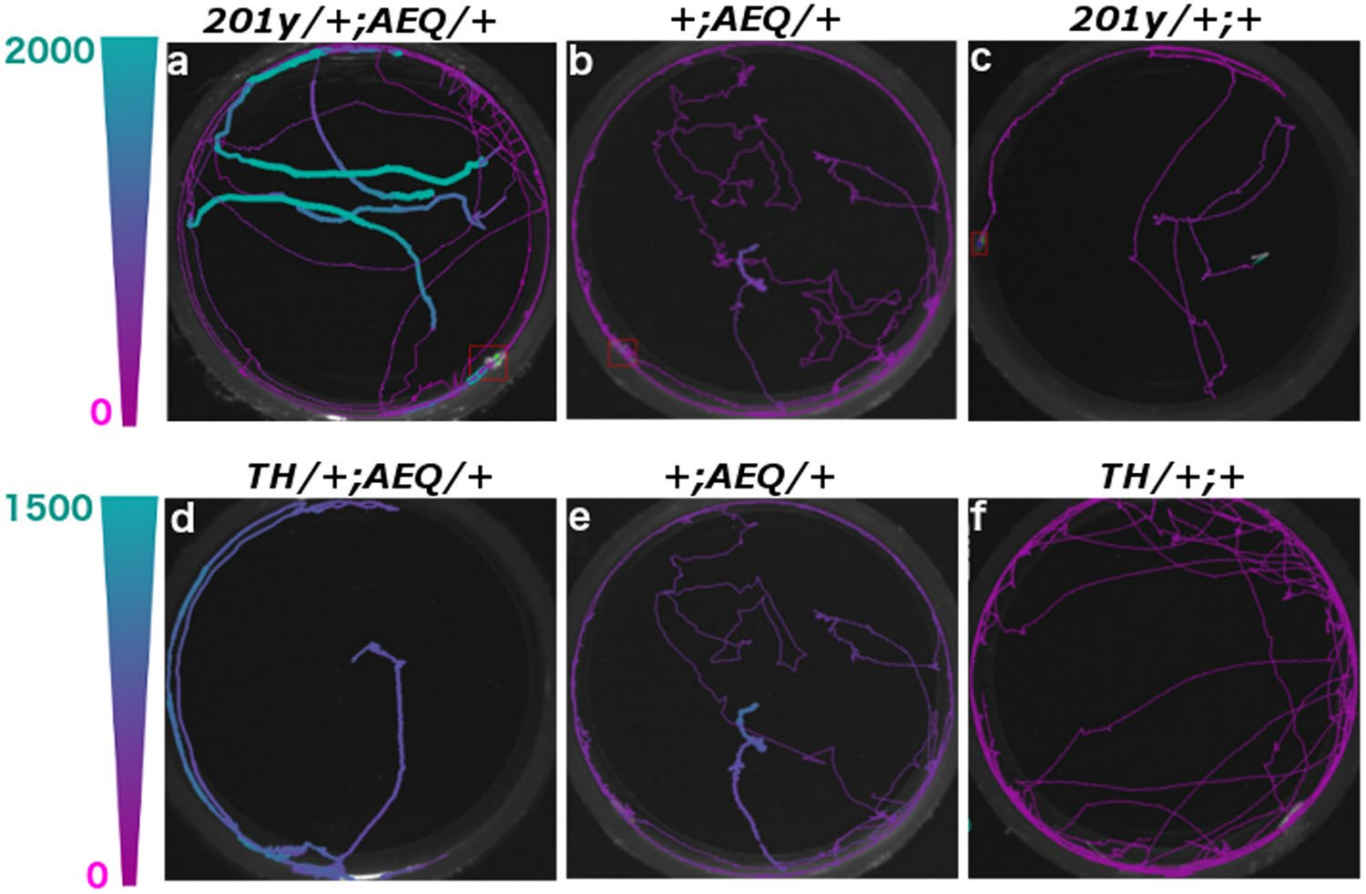
Kenyon cells and Dopaminergic neuron show spontaneous activity. Representative images of larval trajectories with bioluminescence value colour-coded according to heatmap (max. set to 2,000 au in (**a**–**c)**, and to 1,500 in (**d**–**f)**). The line width is increased to enhance contrast between high and low values. (**a**–**c**) 201y-targeted KCs show higher activity compared to the +;*AEQ/*+ (n = 6) control, the same is observed in TH-targeted DAs’ (controls: +;*AEQ/*+ and *TH/*+;+) (**d**–**f**). Bioluminescence frame length = 2,000 ms. Video frame = 50 ms.

We extended this approach to monitor neural activity and behaviour from freely-crawling larvae by constructing a recording apparatus (Fig. 3) similar to one previously described for zebrafish by Nauman *et al*.^11^. There, fast shuttering was used to isolate the PMT from the lighting necessary to gather video data. Here we utilized IR frustrated total internal reflection illumination^23^. This method allows for observing larvae behaviour in light invisible to them and, due to the minimal scattered light, with high contrast. Combined with optical filtering it allows the isolation of the PMT from wavelengths other than the blue spectrum luminescence signal (see Methods). For this protocol, we fed the 625 μM CTZ dose and were able to record spontaneous neural activity from larval KCs ( *201y;AEQ/TM6B*, n = 16) (see Fig. 4 for a sample trajectory) and from TH-targeted DA neurons (*TH/*+; *AEQ/*+, n = 6) (see sample on Fig. 4e) whilst a larva was roaming freely in the recording chamber.

### Recording luminescence from genetically-targeted neurons in response to a stimulus

Going beyond recording spontaneous activity in static environments, one can then aim to observe neural and behavioural changes in response to more dynamic environmental stimuli.

We demonstrated this potential by designing a chamber able to deliver an olfactory cue (see Fig. 3a–c), and tested it using a salient stimulus; one that elicits a well-characterised behaviour and a strong neural response, to show that both of these can be recorded simultaneously by this system.

We chose CO_2_ as a salient olfactory stimulus since it is known that *Drosophila* larvae robustly stop crawling in response to a sudden increase in CO_2_ levels^24–27^. Moreover, a strong calcium influx has been reported in α/β and γ lobe-KC of *Drosophila* adult brains in response to CO_2_^28^. Thus, we postulated that it may be possible to measure higher calcium ion levels using the 201y driver in CO_2_ stimulated larvae, while the simultaneous stop behaviour should also be observable in our video recordings. CO_2_ has the additional advantages that it can be easily obtained from a cylinder at a fixed concentration, and it does not contaminate the plastic chamber, thus simplifying issues associated with clearing the odour after stimulation. Moreover, the CO_2_ concentration in the recording chamber can be measured by mounting a low-cost sensor (Fig. 3b,c), so that the stimulus, the behaviour, and neural responses can be correlated.

**Figure 5:**
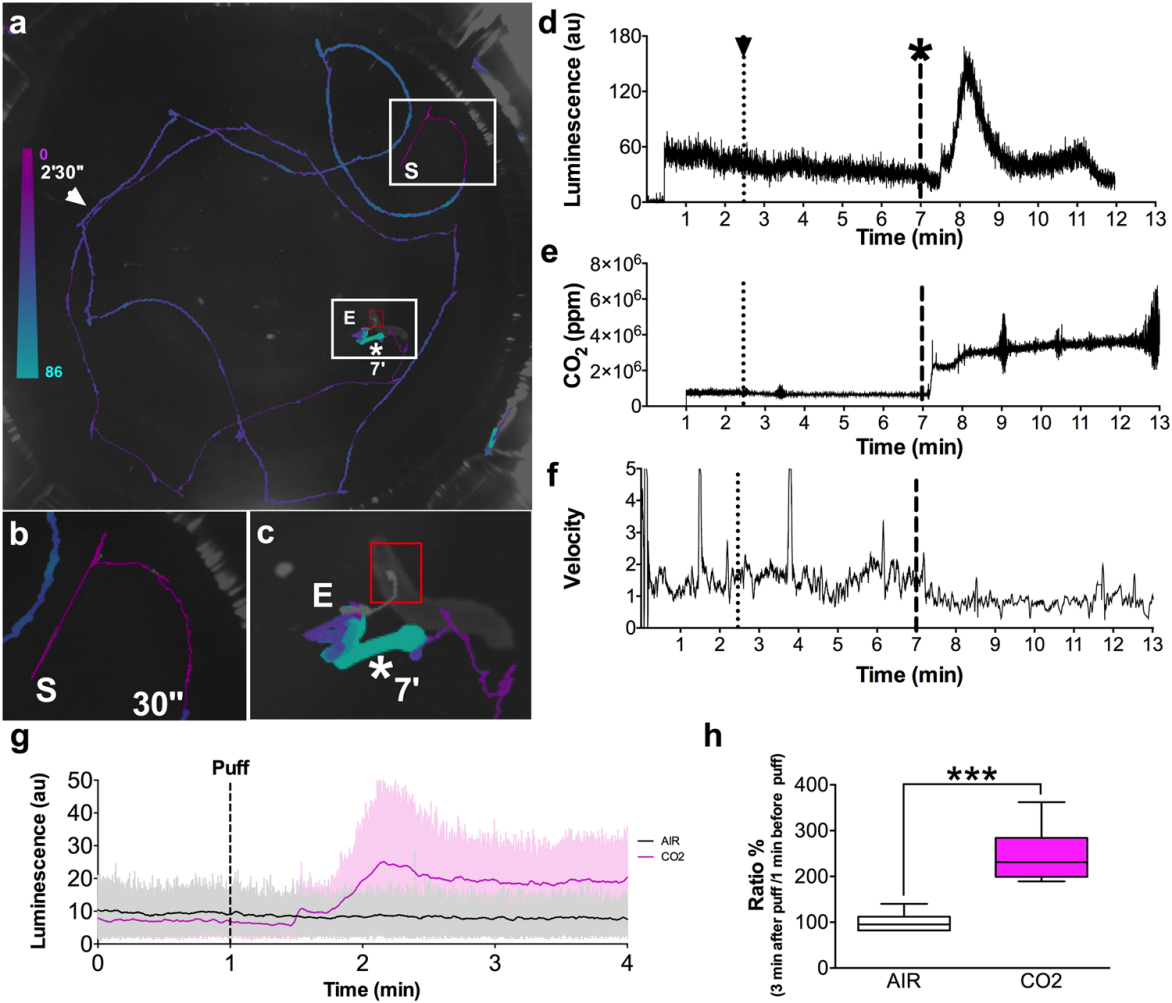
Simultaneous recording of behavior and bioluminescence in comparison to stimulation. One  *201y;AEQ* larva was left to freely move on the arena for 13 min., during which we recorded bioluminescence, CO_2_ levels and a video. At 30 sec. the shutter was opened, at 1 min. the CO_2_ sensor was switched on, at 2 min. 30 sec. (arrowhead) 200 ml air was pumped slowly into the chamber, and at 7 min. (*) 200 ml CO_2_ was pumped slowly into the chamber. **(a)** Example image of one larval trajectory. The line is coloured according to the displayed heatmap. **(b)** Enlarged image of the initial part of the experiment during which the shutter is closed and, therefore, the luminescence signal is low. **(c)** Enlarged image of the larva and trajectory during CO_2_ stimulation. Here the carbon dioxide leads to high bioluminescence and the animal stops. S = start and E = end. The red square identifies the final point of the trajectory. (**d**–**f**) Aligned plots showing neural activity **(d)**, stimulus **(e)** and behaviour **(f**) recorded during the experiment imaged in **(a**–**c)**. **(d)** The trace shows evidence of prior-light PMT contamination, characteristically seen as an initial background signal that slowly decays. Yet, our abilility to record the strong signal seen here following CO2 exposure is not affected. **(g)** Comparison of the mean signal (n = 7) with S.E.M. of the luminescence emitted by larval KCs synchronizing the “puff” moment. The grey line represents the luminescence before and after the “AIR puff”, while the magenta line represents the luminescence before and after the “CO_2_ puff”. **(h)** The CO_2_ puff leads to an increase in the luminescence signal (μ = 244 ± 23%), while the Air puff to no change (μ = 101 ± 8%). Bioluminescence and CO_2_ frame length = 100 ms. Video frame = 50 ms.

We adapted the chamber design of our apparatus to enable the flow of a gas stimulus through an inlet and an outlet channel, and the measurement of CO_2_ levels via a sensor positioned in the chamber above the arena (Fig. 3b,c). Use of the inlet channel alone permitted accumulation of the CO_2_ in the chamber, thus producing a sustained increase reminiscent of a step stimulus (see Fig. 5e).

Using this new setup we conducted experiments on individual larvae (n = 7,  *201y;AEQ*; n = 7,+; *AEQ*; n = 5, Supplementary Figs 5, 7 and 8) to which we delivered either Air or CO_2_ while they were freely crawling in the arena. As expected, a sudden increase in CO_2_ concentration caused larvae to stop crawling, while sustained elevated concentrations led to narcosis/paralysis in all larvae. The CO_2_-induced stops were followed by a marked transient increase in the bioluminescence emitted by the 201y-KCs (Fig. 5). Although the CO_2_ rise time was in the order of seconds, the rise of bioluminescence was slower (>1 minute to peak bioluminescence value), and adapted back to baseline within a similar time-scale. We repeated this experiment using  *201y;AEQ*/*TM6B* larvae, but only observed the same marked increase in luminescence in 2/4 larvae tested, which may suggest that more than a single copy of AEQ is required to achieve sufficient sensitivity for this genotype.

As the KC response to sustained CO_2_ has not been previously reported in larvae, we proceeded to validate our bioluminescence signal by replicating our findings using standard functional imaging. We targeted the expression of *GCaMP6s* to the same KCs population (n = 4,  *201y/*+; *GCaMP6s/*+). Following sustained CO_2_ stimulation, we recorded an increase in fluorescence with a similar profile and evolution to the photoluminescence recorded under similar conditions (Supplementary Fig. 9). This verifies that the KCs response to CO_2_ stimulation is not an artifact of the AEQ calcium-imaging method used. Although similar responses have been previously reported in the adult^28^, it is still unclear if this slow response is due to olfactory input from Gr63a expressing olfactory sensory neurons (OSNs), since odour responses in KCs have been previously described as sparse-and brief^29^. The Ca^++^ changes could perhaps reflect changes in excitability due to CO_2_ induced changes in pH, which have been previously reported in hippocampal slices^30,31^.

## Discussion

We have presented here the first method that enables monitoring of neural activity in intact and unconstrained *Drosophila* larvae while their behavior is simultaneously recorded. We demonstrate the feasibility of detecting the calcium signal reported by an AEQ based bioluminescence from intact larvae. Using constrained animals, we have assessed the relationship between the number of neurons targeted and the bioluminescence emitted, and between the bioluminescence and CTZ administered. We have further demonstrated that neural and behavioural activity can be simultaneously recorded from a freely moving animal, while allowing changes to be observed  in response to a controlled stimulus.

There is clearly scope for further refinement and evaluation of this paradigm, as we discuss in more detail below. We first note some key advantages and usage scenarios. The method can directly exploit the increasingly accurate methods for genetic targeting of specific neurons in the *Drosophila* larva to monitor identified neurons, without requiring expensive imaging tools. The whole system is affordable since it combines off-the-shelf equipment with a few custom elements that can be 3D printed, which allows for adaptations in the setup to fit new experimental requirements. For example, the dark-chamber used to stimulate the larva with CO_2_ can be used to convey other volatile molecules (e.g. odours) or its design can be modified to apply mechanosensory stimulation. Further, such a system can complement a lab’s imaging toolset by being utilised during the experimental design phase to efficiently conduct the initial screening of neural responses, either by recording individual larvae separately, or en masse.

The further integration of behavioural monitoring and stimulation methods is one direction for future development that would improve the system’s usability. Currently, the recordings from the PMT and video data were obtained separately and only fused during post processing on our tracking software. Improved tracking and recording software could integrate video, PMT luminescence, and sensor data in live view during experiments, while also taking care of synchronizing video to luminescence, e.g. by triggering an electromechanical shutter (rather than the manual shutter used here).

There are also several approaches that could be taken to improve the overall sensitivity of the system. The signal to noise ratio can be compromised by contamination of the PMT by light, either during or before a recording is initiated. Indications of the effect of prior light-exposure can be found in some of our data (see Fig. 5d and also Fig. 4e), seen as an initial elevated background signal that slowly decays. As it stands, these issues can be avoided if working under low-light conditions, or in a dark room, and by ensuring proper shutter closure at all times before the light-proof box is opened. Mounting a commercially available shuttering system, that can also be operated from outside the light-proof box, could minimize the risk of contamination further. A complementary approach to increasing the sensitivity, is to further explore the potential of AEQ/CTZ system to increase the signal and reduce variability. For example, we found that using a water-soluble form of the prosthetic group^19^, increased the sensitivity of the system without affecting larval viability.

Ideally, we would like to optimize the approach so as to be able to record from single neurons, which in a small system, such as *Drosophila* larva, may have activity correlated to behavioural changes. In this assay, we demonstrated activity recording from a relatively large group of neurons by taking advantage of the strong KC response to CO_2,_ verifying that the bioluminescence response to CO_2_ is indeed relevant to KC activity by replicating a variant of this experiment using GCAMP imaging on constrained animals. Imaging revealed that the signal is due to the sum of synchronous transient bursts across a large population of the imaged KCs. However, macroscale recordings over large neural populations, with this system, may fail to capture other sparse pattern changes of activity, since it may be possible for the mean luminescence to appear constant although the underlying neural pattern of network activity is modified. As it stands, when recording from small neural populations (1–10 neurons) the signal can be strengthened via increasing the number of larvae in the chamber, but obviously this does not allow to correlate the signal emitted by single larvae to their behaviour. Hence, obtaining a signal from smaller neural populations during free behaviour of an individual animal is a target for future research.

## Methods

### *Drosophila* genetics

Flies were kept at 25 °C on common cornmeal-yeast-sucrose food under light:dark cycles and 60–70% humidity. *UAS-GFP-Aequorin* strain was obtained from Martin *et al*.^14^. Expression of *GFP-apo-Aequorin* was driven to KCs and DAs by *201y-gal4* and *mb247-gal4*, and *TH-gal4* and *R58E02*, respectively. Either the driver and transgene lines were crossed to *CS*^*w−*^ strain to generate larvae to use as genetic controls of the experiments. Also, *mb247-gal4;UAS-tubP-Gal80*^*ts* ^^32^ was crossed to either *UAS-GFP-Aequorin* strain or to control the specificity of the KC signal. Expression of *20XUAS-IVS-GCaMP6s* (42749, Bloomington Stock Center) was driven to the KCs by *201y-gal4*.

### Aequorin reconstitution

For reconstitution with CTZ, 3-day-old larvae were incubated for two days with cornmeal-yeast-sucrose food containing a final concentration of 12.5–625 μM water soluble CTZ (Prolume), depending on the experiment (see text). The incubation with feeding, of the above mixture, was extended to three days for the recordings on intact and freely behaving larvae. All CTZ stock solutions consist of 12.5 mM dissolved in MilliQ water and they are kept at −20 °C.

### Bioluminescence analysis of intact “partially constrained” larvae

On the day of analysis, larvae were briefly washed with MilliQ water, and then, transferred into 100 μl of MilliQ water inside a transparent vial (∅ = 1 cm and height = 4 cm). This vial was then placed into a light-proof recording chamber equipped with an Electron Tubes Enterprises 9111B80 photomultiplier (PMT) that records luminescence (see Supp. Fig. 1(a–e)). The total bioluminescence emitted was recorded. Each bioluminescence trace represents a single larva. The capture rates used were 2,000 ms or 100 ms depending on experiment. Arbitrary units (au) are used to represent the photon counts that represent the bioluminescence amplitude as read from the PMT.

All data were analysed using GraphPad 6 software. One-way or Two-way analysis of variance was adopted to carry out the statistical analysis (Sidak test).

### Design of the apparatus for bioluminescence analysis of intact and freely-behaving larvae

A light-proof enclosure was built to house all the system components of the bioluminescence setup (Fig. 3a). A camera, which had its IR filter removed, was placed under the arena to record larval movement, a PMT was mounted above the arena. Between the PMT and the camera sit three custom made units that were 3D printed using black PLA plastic (Fig. 3) and serve the following purposes: (1) to illuminate the larva with IR light such that it can be seen by the camera positioned underneath. (2) To reduce any light-contamination of the PMT, from both the IR lights, leakage of the light-proof box but critically to shutter-off environmental light when the light-proof box is opened between experiments. For this, a 3 mm gauge metal rod was attached to the rear of the shutter that extended to the outside of the light-proof box, through a hole on the back, so that the shutter can be operated externally, before the light-proof box is opened; (3) to constrict the larva to move within the PMT’s field of view, where the effect of relative position to luminescence is minimized.

Lighting was provided by a ring of IR LEDs arranged around the base of our custom design Petri dish holder, so as to illuminate the agarose from the side. This simple technique is our implementation of a type of dark field illumination, utilizing frustrated total internal reflection recording method^23^, which increases image contrast and reduces the light contamination of the PMT by trapping most light via total internal reflection, except for light scattering where the larva touches the substrate.

The sensitivity of the PMT falls, rapidly, towards the red, but nevertheless the IR light can still contaminate the background signal of recordings. Given that the wavelength of AEQ is in the blue end of the visible spectrum, we added a combination of optical filters on our shutter’s design at the open position which allowed us to filter out IR and permit only the luminescence wavelength, thus increasing our signal to noise ratio.

### Bioluminescence analysis assay of freely-behaving larvae

Before analysis, larvae were briefly washed into MilliQ water, as described above. Then, they were placed on a circular arena filled with a thin layer of 4% pure agarose (Bioline Cat.BIO-41025). To analyse KCs and TH-DAs, single *Drosophila* larvae were placed on the behaviour plate during the test. A different larva was used for each test. At least four tests were run for each genotype.

The videos of the larval crawling were recorded at 20 fps. The extraction of the larval trajectory and speed, and the overlay of the video-synchronized luminescence signal, were performed using a custom prototype tracking software, which utilizes the OpenCV^33^ library for image processing and background segmentation so as to identify and log the position of the larvae at every frame to a file. The tracker also allowed to load luminescence data and draw it as a colour heatmap on the larva’s trace. The track data files, are simple text files that can be imported into a data processing software for further analysis.

To synchronize the signals between PMT and video we utilized the effects of opening our manual shutter. Opening of the shutter caused as a sudden change in BG signal as luminescence was allowed through, while painting of a white dot on the shutter allows for detecting the time when the shuttered is opened in the video. Further, there is a framerate difference between the PMT log and the video frame rates which was accounted for in our tracking software via a scaling factor so that the luminescence sampled timebase follows the video frame number.

### CO_2_ larval stimulation set-up for bioluminescence analysis

We designed and 3D printed a new dark chamber, out of black PLA plastic, that has an inlet/outlet pipe configuration, so as to allow influx and build-up of CO_2_ concentration_,_ and its subsequent clearance via forced air-flow through the inlet and outlet pipes (Fig. 3b,c). The 3D models are available for download^34^. The gas/air flow is diffused in the chamber by forcing the flow via numerous tiny holes on the chamber walls. The chamber was also designed to accommodate a CO_2_ sensor, which allowed us to correlate changes in CO_2_ with neural activity, but also to control for clearing the chamber with clean air-flow between experiments.

CO_2_ was delivered through one of the pipes, found on either side of the chamber (Fig. 3b). The clearing airflow was generated by disconnecting CO_2_ input and forcing airflow via a vacuum pump connected to the pipe on the other side the chamber. This was activated after every analysis for a sufficient amount of time until the CO_2_ levels, as monitored by an integrated CO_2_ sensor, returned to atmospheric levels. The CO_2_ concentration in the analysis chamber was monitored using a widely available and affordable solid electrolyte cell CO_2_ sensor, the MG811. This came as module composed of sensor and analog signal amplifier. The module encodes CO_2_ levels in a voltage range of 0–2 V. An Arduino nano was connected to the sensor module to perform analog to digital conversion and to send the readings to a desktop computer. The sensor was calibrated using the MG811 datasheet’s sensitivity chart and assuming atmospheric air is at 400 ppm.

Supplementary Table 1 shows the approximate costs of the setup used for the system we present in this manuscript.

### Functional imaging

Second-instar larvae were placed on a nitrocellulose-covered glass slide and were wounded at the posterior third of the larvae, so as to restrict movement, leaving the anterior body intact. The body was bathed in a solution containing 115 mm NaCl (Ambion), 5 mm Na-HEPES (Sigma), pH 7.5, 1 mm MgCl_2_ (Ambion), 5 mm KCL (Fisher Scientific), 6 mm CaCl_2_ (Sigma), 4 mm NaHCO_3_ (Sigma), 10 mm glucose (Sigma), and 65 mm sucrose (Alfa Aesar). A coverslip was placed on top of the larvae to further minimize the animal’s movement. The coverslip was held in place by Vaseline applied to each corner^35^. CO_2_ was delivered to the head of the larvae through a steel 27 gauge needle connected to plastic tubing. A gas cylinder with a flow regulator was used to deliver CO_2_ at sufficiently low flow rates such that gas flow does not disturb the larva from its placement under the objective. To control for multiple factors that could contribute to getting no response (larvae severely wounded, or olfactory organ being occluded by the bath or cover slip as the animal moves), in our analysis we only include larvae that produced any KC response to the CO_2_ stimulus and report the average response of these.

### Immunostaining of larval brains

Larval brains from 5-days-old larvae were dissected in ice-cold Schneider’s (S2) medium and fixed in 2% PFA in S2 overnight at 4 °C. Fixative was washed away with PAT3 solution. The tissues were blocked with 3%NGS-PAT3 solution for 2 h at RT. Primary antibody used were: rabbit anti-GFP (A11122, Invitrogen) at 1:5000 dilution and nc82 (University of Iowa Hybridoma Bank, Iowa City, IA) at 1:20 dilution overnight at 4 °C. Secondary antibodies used were: Goat α-Rabbit Alexa Fluor 488 (A11008; Invitrogen) and Goat α-Mouse Alexa Fluor 546 (A21123; Invitrogen) both at 1:1000 dilution for 5 days at 4 °C. Brains were mounted and images taken according to Young JM and Armstrong JD^36^.

### Ethical approval and informed consent

The experiments reported in this manuscript were not carried out on either live vertebrates or human samples.

## Electronic supplementary material

Supplementary information
