# Supplementary material for: Monitoring brain activity and behaviour in freely moving Drosophila larvae using bioluminescence
Source: Sci Rep. 2018 Jun 18;8:9246. doi: 10.1038/s41598-018-27043-7 (PMC6006295; doi:10.1038/s41598-018-27043-7)
Supplement: Supplementary file 1 — Supplementary information [file 41598_2018_27043_MOESM1_ESM.docx]

**Monitoring brain activity and behaviour in freely moving *Drosophila* larvae using bioluminescence**

Marescotti Manuela^1^*^§^, Lagogiannis Konstantinos^2,3§^, Webb Barbara^2^, Davies R. Wayne ^1,2^, and Armstrong J. Douglas ^1,2^

^1^ Brainwave-Discovery ltd.

^2^ The University of Edinburgh

^3^ Centre Of Developmental Neuroscience, King’s College London

*Corresponding [m.marescotti@brainwave-discovery.com](mailto:m.marescotti@brainwave-discovery.com)

^§^ These authors contributed equally to the work

**Supplementary Information**

**Supplementary Figure 1:** Overview of the apparatus used to record neural activity from intact *Drosophila* larvae suspended in vial tubes. **(a)** A light-proof box contains the main components: the PMT is mounted on a cylindrical metal casing which has a hole for inserting a vial (Ø = 1 cm and hight = 4 cm). Once inserted the vial is moved into position in front of the PMT via a rotor **(b-d)**. The PMT collects the bioluminescence from the side of the vial. **(e)**The vial contains the larva submerged in 100 μl of MilliQ water.

**
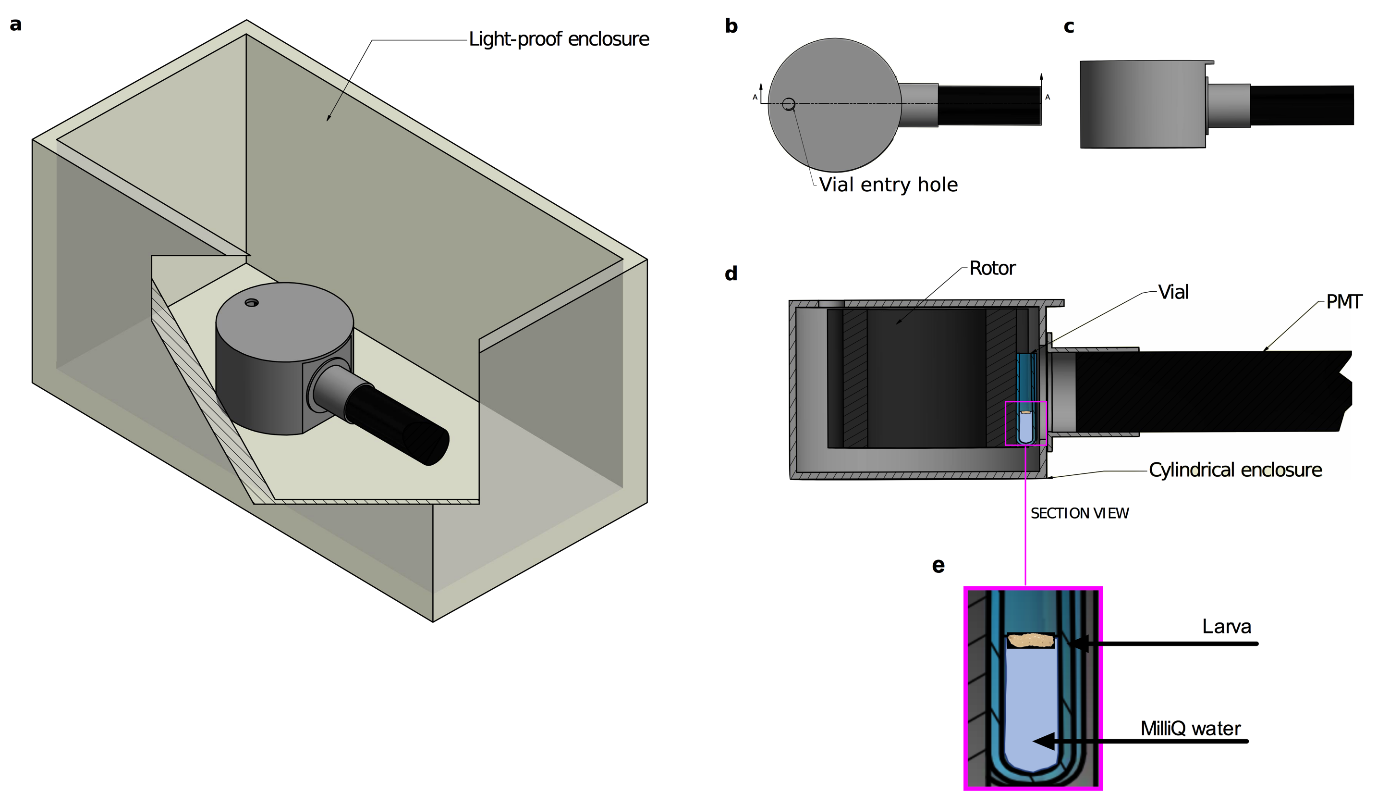
**

**Supplementary Figure 1**

**Supplementary Figure 2:** Spontaneous neural activity from larval KCs **(a)** Bioluminescence recorded from *mb247/+;UAS-Tub-gal80^ts^/+;AEQ/+* larvae (n = 10 , μ = 136.116 au ± 2.178 au) is compared to that detected from *201y/+;AEQ/+*, *mb247/+;AEQ/+* and *mb247/+;UAS-Tub-gal80^ts^/+;+* larvae (n = 3, μ = 109.860 au ± 3.307 au). **(b)** Representative traces from *mb247/+;AEQ/+* larvae showing variable activity. **(c)** Representative trace showing the luminescence emitted by *mb247/+;UAS-Tub-gal80^ts^/+;AEQ/+* larvae. Bioluminescence frame length = 2,000 ms. *****p<0.0001*

**

**

**Supplementary Figure 2**

**Supplementary Figure 3:** Bioluminescence signal amplitude depends on the CTZ concentration. Mean signal recorded over 30 minutes from *201y/+;AEQ/+* using 12.5 μM (n = 28, μ = 1,065.989 au ± 57.085 au), 125 μM (n = 12, μ = 46,747.210au ± 3,038.160au) or 625 μM CTZ (n = 12, μ = 74,243.3 au ± 4,325.243 au). (*****P<0.0001*).

**

**

**Supplementary Figure 3**

**Supplementary Figure 4:** Expression patterns of R58E02-targeted Dopaminergic neurons and their bioluminescence signal according to the CTZ concentration. **(a-c)** R58E02 crossed with UAS-*GFP-aequorin* and double stained by anti-GFP (green) and nc82 (neuropile marker; magenta). Scale bar represents 100 μm. **(d)** Mean signal recorded over 30 minutes from *R58E02/+;AEQ/+* larvae (n = 9, μ = 142.95 au ± 3.75 au) using 12.5 μM, and from *R58E02/+;AEQ/+* larvae (n = 6, μ = 198.35 au ± 5.48 au using 625 μM CTZ. Sidak’s multiple comparison test was performed between the *R58E02/+;AEQ/+* and the two genetic controls for both 1XCTZ and 50XCTZ group of samples. (**e**) Representative traces showing the luminescence emitted from one *R58E02/+;AEQ/+* larva fed with 625 μM CTZ*.* (*****P<0.0001*). Bioluminescence frame length = 2,000 ms.


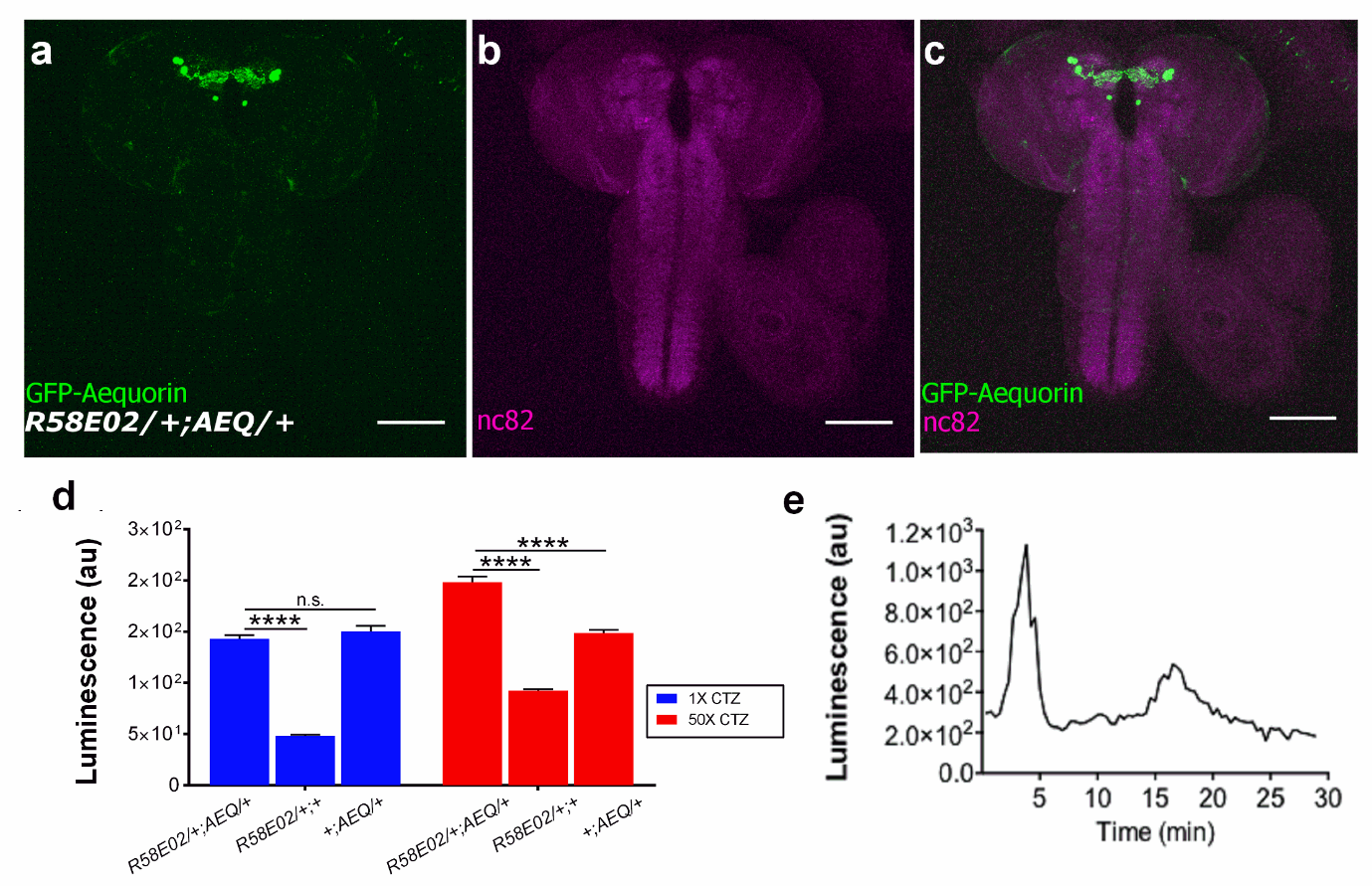


**Supplementary Figure 4:**

**Supplementary Figure 5:** Single traces showing the luminescence emitted from *201y;AEQ* larvae in response to either Air or CO_2_ puff.

**
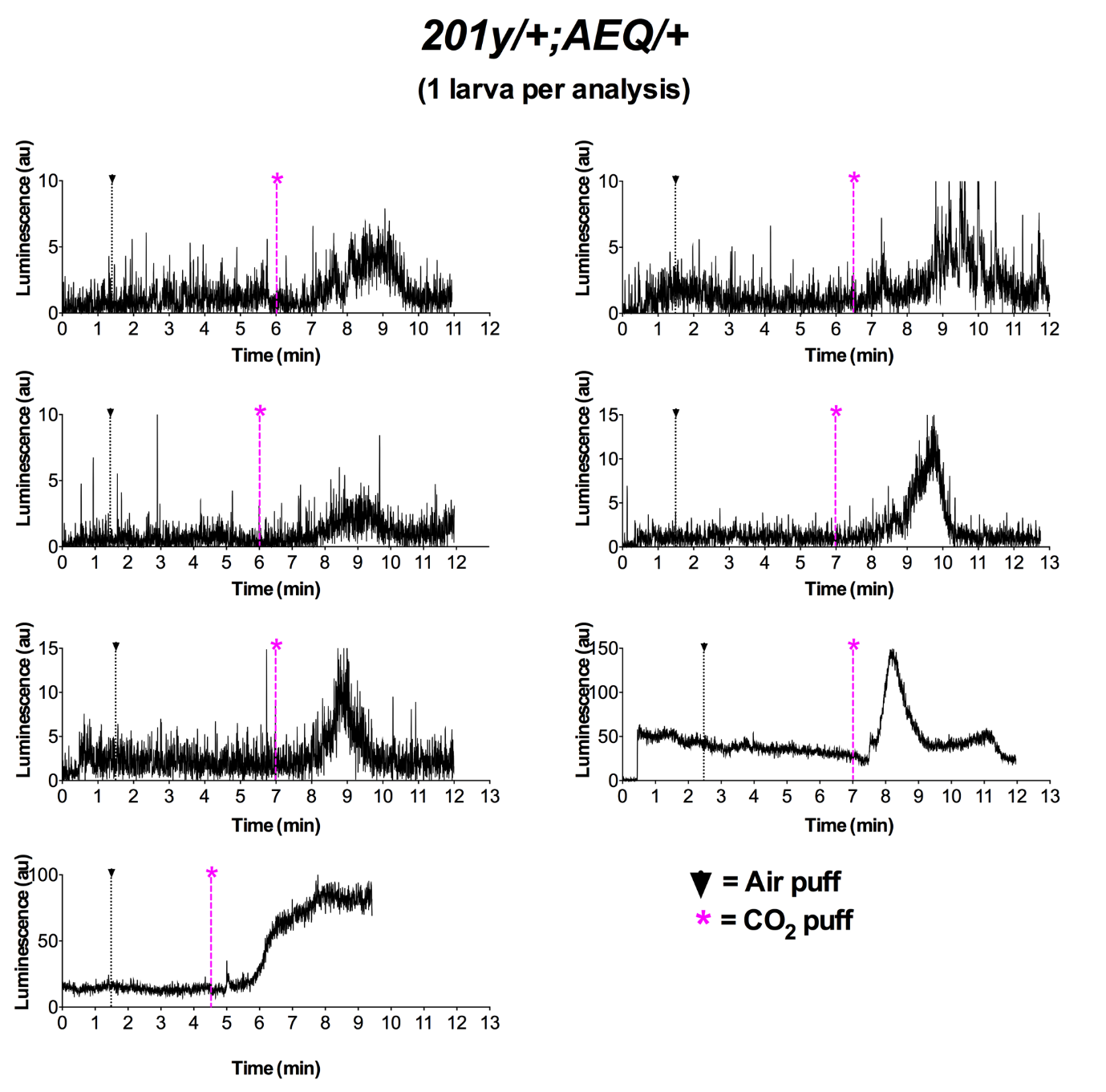
**

**Supplementary Figure 5**

**Supplementary Figure 6: (a)** Comparison of the mean signal (n = 7) of the luminescence emitted by larval KCs synchronizing the CO_2_ puff. The grey line represents the mean luminescence with S.E.M emitted by *+;AEQ/+* larval KCs while the magenta line the mean luminescence with S.E.M emitted by *201y;AEQ* larval KCs. **(b)** The CO_2_ puff leads to an increase in the luminescence (μ = 244% ± 23%) emitted by *201y;AEQ* larval KCs, while it does not change (μ = 94% ± 6%) the luminescence emitted by *AEQ/+* larval KCs. Bioluminescence frame length = 100 ms.

**
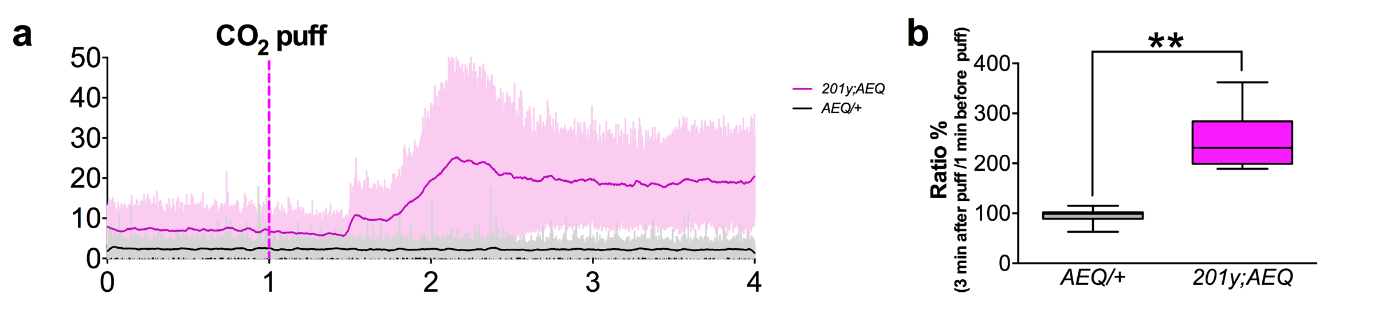
Supplementary Figure 6**

**Supplementary Figure 7:** Single traces showing the luminescence emitted from *+;AEQ* larvae in response to CO_2_ puff.

**
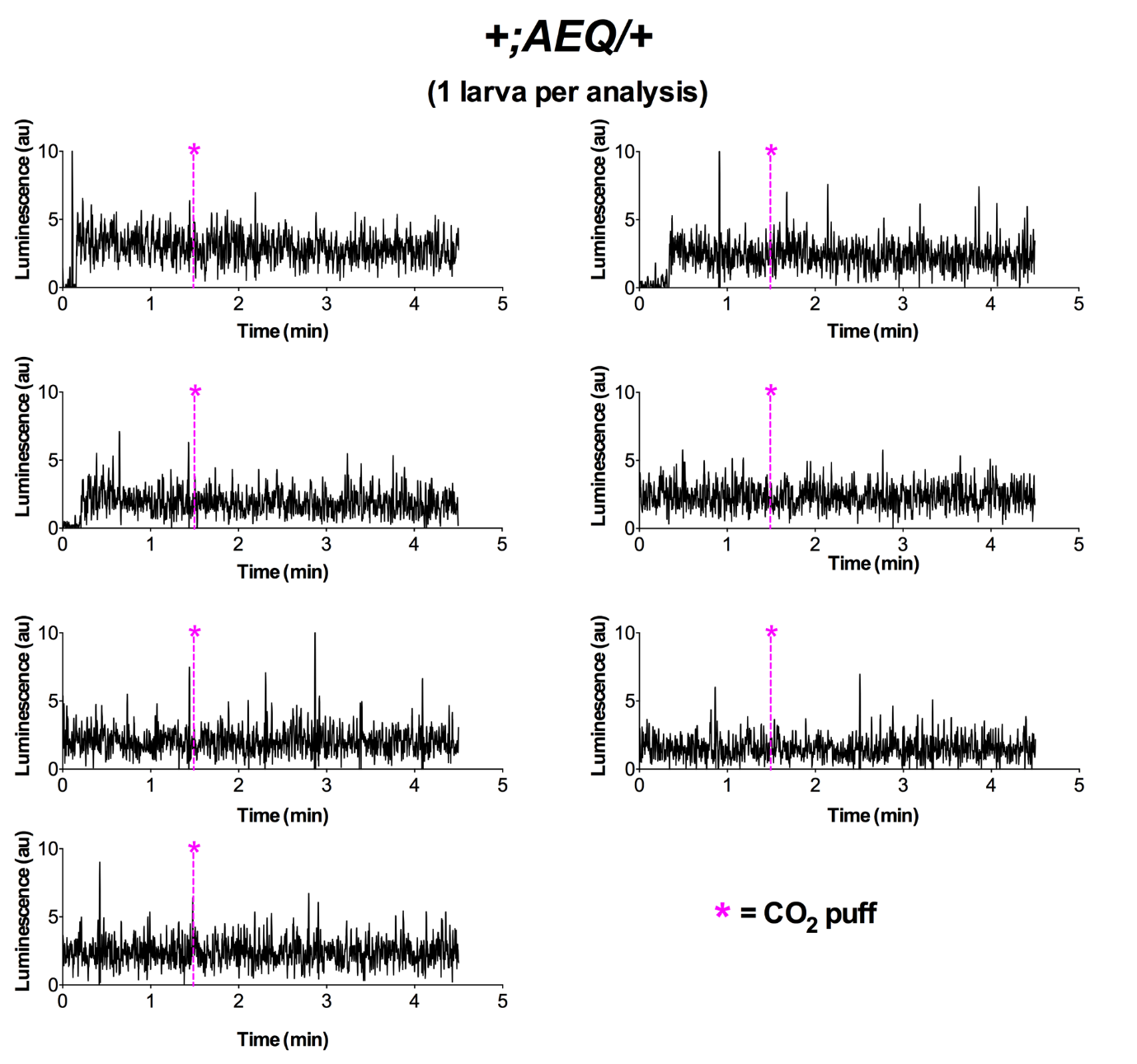
**

**Supplementary Figure 7**

**Supplementary Figure 8:** Single traces showing the luminescence emitted from *201y/+;+* larvae in response to CO_2_ puff.

**
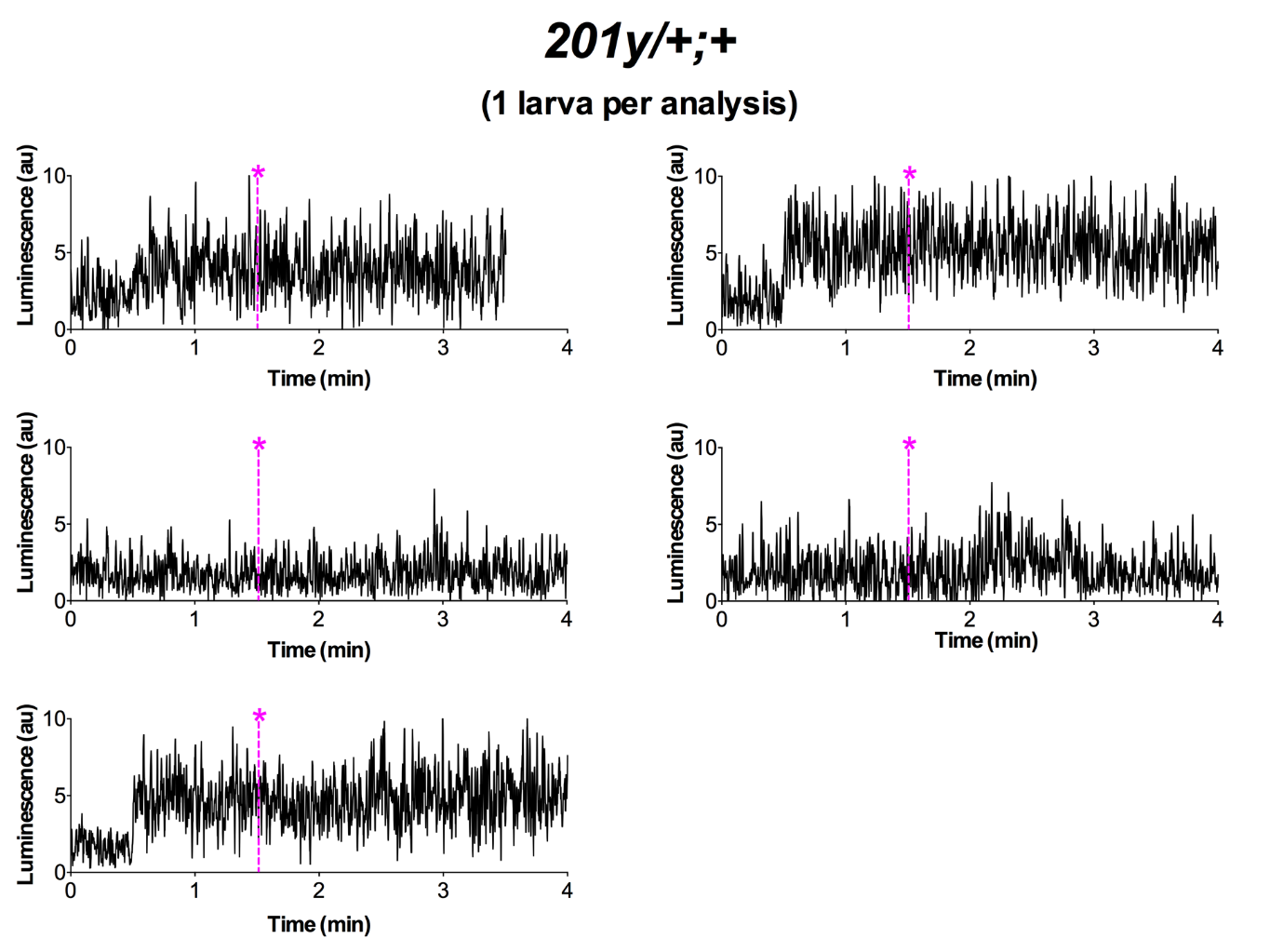
**

**Supplementary Figure 8**

**Supplementary Figure 9:** Mean signal (n = 4) of the fluorescence emitted by larval KCs of *201y/+; GCAMP6s/+ larvae* synchronizing the CO_2_ puff.

**

**

**Supplementary Figure 9**

| **Item** | **Approximate Cost (£)** | **Notes** |
| --- | --- | --- |
| Photomultiplier Tube | 950 | ET enterprises Electron Tubes |
| 3-D printed parts | 100 |  |
| 2xMetallic rods | 30 | MTTS |
| Camera + lenses | 700 | Pointgrey |
| Optical Filter | 50 | ThorLabs |
| IR LEDS | 5 | Bright Components |
| Dual channel power supplier | 200.00 |  |
| CO2 sensor +Arduino | 40.00 |  |
| Enclosure | 20 | Wooden sheets and metallic rods b&q |
| **Total** | 2095 |  |

**Supplementary Table 1. Table of approximate setup costs**
